# Efficacy of Magnetic Sphincter Augmentation versus Nissen Fundoplication for Gastroesophageal Reflux Disease in Short Term: A Meta-Analysis

**DOI:** 10.1155/2017/9596342

**Published:** 2017-03-30

**Authors:** Ming-yu Chen, Di-yu Huang, Angela Wu, Yi-bin Zhu, He-pan Zhu, Liu-mei Lin, Xiu-jun Cai

**Affiliations:** ^1^Department of General Surgery, Sir Run Run Shaw Hospital, College of Medicine, Zhejiang University, Hangzhou, Zhejiang, China; ^2^Medicine, University of Melbourne, Melbourne, VIC, Australia

## Abstract

*Background*. The efficacy of Magnetic Sphincter Augmentation (MSA) and its outcomes for Gastroesophageal Reflux Disease (GERD) are uncertain. Therefore, we aimed to summarize and analyze the efficacy of two treatments for GERD.* Methods*. The meta-analysis search was performed, using four databases. All studies from 2005 to 2016 were included. Pooled effect was calculated using either the fixed or random effects model.* Results*. A total of 4 trials included 624 patients and aimed to evaluate the differences in proton-pump inhibitor use, complications, and adverse events. MSA had a shorter operative time (MSA and NF: RR = −18.80, 95% CI: −24.57 to −13.04, and *P* = 0.001) and length of stay (RR = −14.21, 95% CI: −24.18 to −4.23, and *P* = 0.005). Similar proton-pump inhibitor use, complication (*P* = 0.19), and severe dysphagia for dilation were shown in both groups. Although there is no difference between the MSA and NF in the number of adverse events, the incidence of postoperative gas or bloating (RR = 0.71, 95% CI: 0.54–0.94, and *P* = 0.02) showed significantly different results. However, there is no significant difference in ability to belch and ability to vomit.* Conclusions*. MSA can be recommended as an alternative treatment for GERD according to their short-term studies, especially in main-features of gas-bloating, due to shorter operative time and less complication of gas or bloating.

## 1. Introduction

Gastroesophageal Reflux Disease (GERD) remains a major disease burden [1–3] and ranges from erosive esophagitis to Barrett's esophagus [4]. The heartburn or regurgitation is the most common and progressive main-feature. There are many treatments to control reflux symptoms including proton-pump inhibitors (PPIs), transoral incisionless fundoplication (TIF), and Nissen and Toupet Fundoplication [5, 6]. Although GERD can be treated effectively by PPIs in most patients (approximately 60 percent) [7], timely surgical intervention is necessary for inadequate control of reflux symptoms. However, it does not mean that only patient who suffered from the failure of medical management should undergo surgical intervention [8, 9]. In fact, surgical interventions such as Nissen and Toupet Fundoplication are no worse than PPIs [10–12], but the procedure remains unsatisfied, due to adverse events consisting of bloating and inability to belch or vomit [13, 14]. Consequently, many treatments have been introduced instead of NF. TIF and MSA were approved by the U.S. Food and Drug Administration (FDA) in 2007 [15] and 2012 [16], respectively. MSA is one of the latest methods to treat GERD using LINX system (Torax Medical). However, the present literatures associating with efficacy of MSA and comparing to Nissen surgery are limited and unclear [17–19]. Therefore, it is essentially necessary to get a comprehensive understanding on the difference of efficacy between MSA and NF for GERD.

## 2. Materials and Methods

This meta-analysis was adhered to the guidelines of Preferred Reporting Items for Systematic Reviews and Meta-Analyses (PRISMA).

### 2.1. Study Selection

Two of the authors (Mrs. Wu and Mr. Zhu) performed the meta-analysis search independently, using PUBMED, EMBASE, OVID, and Cochrane database. The search was performed on all studies comparing MSA and NF from 2005 to 2016, and its strategy was based on the following Medical Subject Heading (MeSH) terms: “Magnetic Sphincter Augmentation”, “MSA”, “LINX device” “Nissen Fundoplication”, “NF”, and “LNF”. Only studies on humans and in English and Chinese language were considered for inclusion. Reference lists of all retrieved articles were manually searched for additional studies.

### 2.2. Data Extraction and Conversion

Two authors (Mr. Zhu and Mrs. Liu) were required to perform data extraction, independently and respectively. The parameters for each study included the following: (1) first author, publication year, and study design; (2) the number and characteristics of patients; (3) the outcome of the studies including number or incidences of adverse events and complications (postoperative dysphagia, belch, and vomit) and proton-pump inhibitor use. If available, the RRs with their 95% CIs and *P* values were collected from the original article or the corresponding E-mails. If not, we calculated RRs and their 95% confidence interval using the data of samples in each group or the data provided by the authors. If only Kaplan–Meier curves were available, we extracted data from the graphical survival plots and estimated the RRs. All the calculations mentioned above were based on the methods provided by Tierney and Parmar.

### 2.3. Inclusion Criteria

If trials were included in the meta-analysis, the criteria had to be fulfilled as follows: (1) Compare the original outcomes of MSA and NF for the treatment of GERD; (2) report on at least incidence of adverse events, complications, and proton-pump inhibitor use; (3) if dual studies were reported by the same institution or authors, only the most recent publication or the highest quality of study was included.

### 2.4. Exclusion Criteria

The following trials were excluded: (1) those dealing GERD with second surgery; (2) those using TIF for GERD; (3) those without clear outcomes; and (4) abstracts, letters, editorials and expert opinions, case reports, and studies lacking control groups.

### 2.5. Statistical Analysis

The Review Manager (RevMan, version 5.3) was used to perform this mate-analysis. Proton-pump inhibitor use, the number or incidence of adverse events, and complications (postoperative dysphagia, belch, and vomit) were analyzed using estimation of RR with a 95% confidence interval (95% CI). Either fixed or random effects model was used to calculate pooled effect. The test of heterogeneity of combined RRs was carried out using Cochran's* Q* test and Higgins* I*-squared statistic. If the* I*^2^ statistic was > 50%, we considered heterogeneity to be present and random effects were performed. If It was less than 5% of a chance occurrence (*P* < 0.05), all statistical data were considered significant.

## 3. Results

### 3.1. Selection of Trials

**Figure 1:**
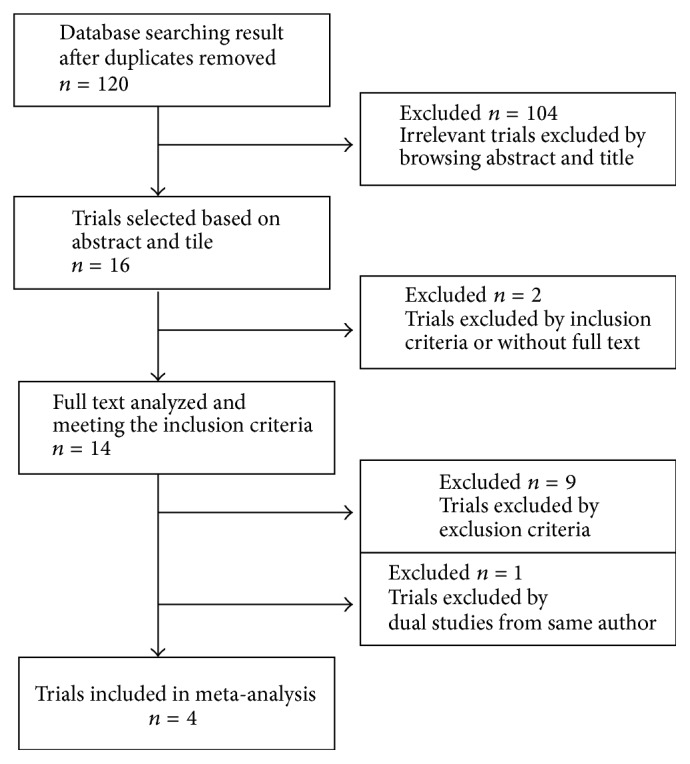
Flow chart showing the selection of studies in the meta-analysis.

Of 14 clinical trials that initially met the inclusion criteria, 8 did not display the specific comparison of the effects of MSA and NF, 1 [20] was reported by one author from the same center, and 1 [21] did not provide enough original data. Finally, 4 [22–25] retrospective studies matched the selection criteria and were published between 2005 and September 2016 (Figure 1). The characteristics of these 4 studies are summarized in Table 1. A total of 624 patients consisted of 299 in the MSA group and 325 in the NF group. The proportion of female (RR = 1.23, 95% CI: 1.05–1.45, and *P* = 0.25), age (RR = 1.69, 95% CI: −1.26–4.64, and *P* = 0.26), and BMI (RR = −1.72, 95% CI: −4.63–1.19, and *P* = 0.25) were not significant, while MSA had a shorter operative time (RR = −18.80, 95% CI: −24.57 to −13.04, and *P* = 0.001) and length of stay (RR = −14.21, 95% CI: −24.18 to −4.23, and *P* = 0.005).

### 3.2. Outcomes

#### 3.2.1. Postoperation PPIs Usage

**Figure 2:**
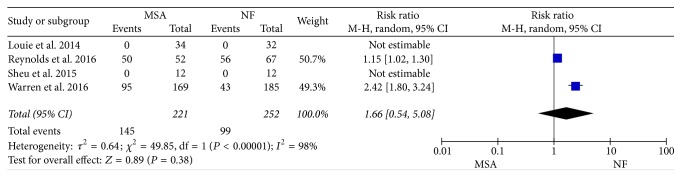
Forest plot of studies evaluating risk ratios of postoperation PPIs usage.

GERD patients who underwent MSA and NF had no significant difference in the resumption of PPIs (RR = 1.21, 95% CI: 0.89–1.65, and *P* = 0.23). There is no heterogeneity among the 4 studies, and a fixed effect model was used (Figure 2).

### 3.3. Complications

**Figure 3:**
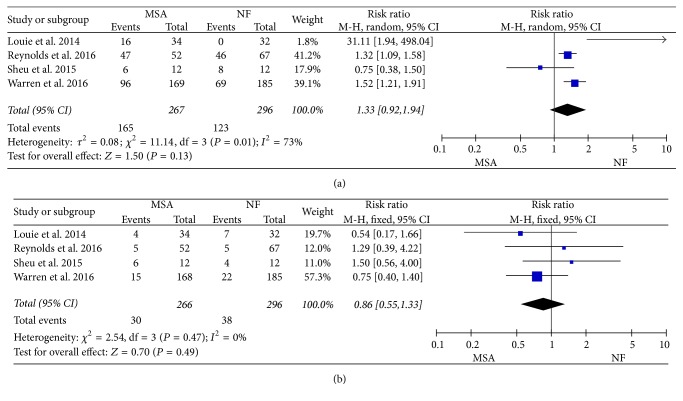
Forest plot of studies evaluating risk ratios of the number of complications (a) and severe dysphagia for dilation (b).

Dysphagia is the most common complication, and severe dysphagia needs second surgery for dilation. The meta-analysis also showed no significant difference of complication (RR = 1.16, 95% CI: 0.93–1.46, and *P* = 0.19) (Figure 3(a)) and severe dysphagia for dilation (RR = 1.36, 95% CI: 0.23–8.02, and *P* = 0.74) (Figure 3(b)) between two groups, when fixed effect and random effect model were used, respectively.

### 3.4. Adverse Events

**Figure 4:**
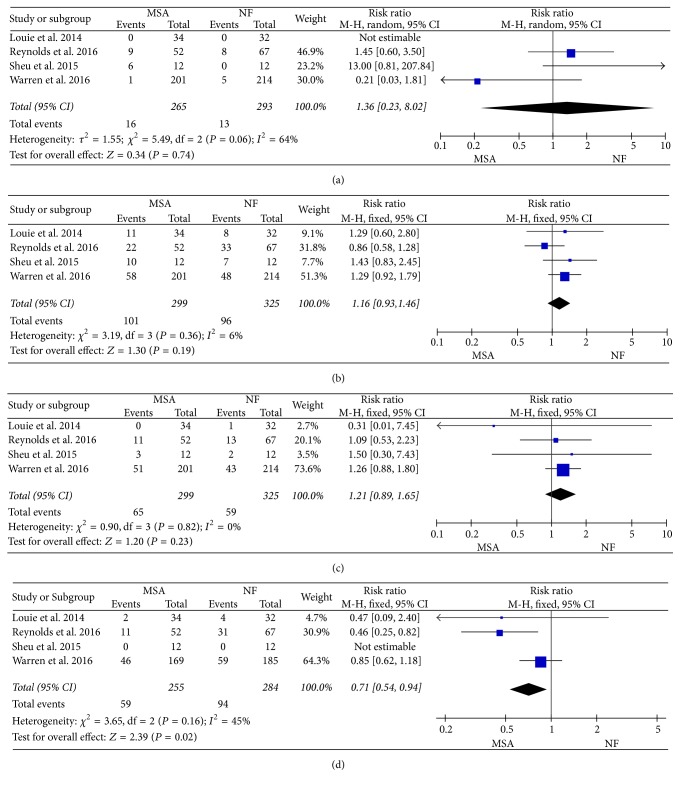
Forest plot of studies evaluating risk ratios of adverse events (a), ability to belch (b), ability to vomit (c), and gas-bloating (d).

No statistical difference existed at incidence of adverse events (RR = 0.86, 95% CI: 0.55–1.22, and *P* = 0.49) (Figure 4(a)). Hence, there is no significant difference in ability to belch (RR = 1.33, 95% CI: 0.92–1.94, and *P* = 0.13) (Figure 4(b)) and ability to vomit (RR = 1.66, 95% CI: 0.54–5.08, and *P* = 0.38) (Figure 4(c)). However, a lower trend toward gas or bloating (RR = 0.71, 95% CI: 0.54–0.94, and *P* = 0.02) was shown (Figure 4(d)).

### 3.5. Publication Bias

Funnel plots were used to perform publication bias of included trials. The funnel plots were almost symmetric. Hence, no evidence for significant publication bias existed in this meta-analysis.

## 4. Discussion

Gastroesophageal Reflux Disease (GERD) caused by the reflux of stomach contents is a major disease burden worldwide [1–3] and severely influences patients' quality of life [26]. Although medical therapy using proton-pump inhibitors (PPIs) can inhibit gastric acid secretion effectively [27, 28], up to 40% of patients still need a surgical treatment for antireflux such as Nissen and Toupet Fundoplication [7]. However, due to the postoperative adverse effects of traditional Nissen Fundoplication, it has not been performed widely [13, 14]. Since Magnetic Sphincter Augmentation (MSA) had been introduced in 2008 and approved by the U.S. Food and Drug Administration (FDA) in 2012 [16], it seems to allow physiologic reflux by restoring a more physiologic sphincter and be regarded as a potential surgical treatment for GERD [22].

MSA is a new alterative surgery with minimally invasive technique and the LINX device encircling the gastroesophageal junction to reduce and control reflux [29]. Nissen Fundoplication is a typical and standard surgery for GERD, especially after the failure of medical therapy. However, it remains a debt whether the efficacy of MSA can come to up expectation in treatment for GERD as similar as NF. In this meta-analysis, MSA had a shorter operative time and length of stay compared to NF, but the similar outcomes in the number of adverse events and complication between two groups were shown. Interestingly, subgroup analysis described some differences on the incidence of postoperative gas or bloating (RR = 0.71, 95% CI: 0.54–0.94, and *P* = 0.02), while there is no significant difference in ability to belch (RR = 1.33, 95% CI: 0.92–1.94, and *P* = 0.13) and ability to vomit (RR = 1.66, 95% CI: 0.54–5.08, and *P* = 0.38). Louie et al. [22] hypothesized that those differences may be explained by restoration of a more normal sphincter, and the gastroesophageal junction improved continually, when MSA is used. Hence, GERD patients can have similar control of reflux symptoms from both of the groups, and MSA has some advantages including shorter operative time and less complication of gas and bloating.

The adverse events and complications consisting of dysphagia, gas/bloating, and inability to belch and vomit occur at low rates (approximately 0.1%) [30] in MSA and NF. Unfortunately, dysphagia remains the most frequent and severe postoperative complication. Of course, the symptom of dysphagia will be better and better in 1 week after surgery in a few patients, due to edema disappearing. If it has no self-resolve over 3 months, timely endoscopic dilation is necessary [16, 17, 31]. Once endoscopic dilation is failed, the MSA devices should been removed.

There are still many unanswered questions whether MSA is still appropriate for hiatal hernias which are more than 3 cm, whether the long-term outcomes of MSA are same as the short-time outcomes, whether the incidence of LINX device removed and erosion will increase as time goes on, and so on. Therefore, it is very important and necessary to perform randomized controlled trials to describe the efficacy of MSA compared to NF in short term and long term.

Limitations of our analysis include two trials which did not match the size of hiatal hernias, the less number of tails included, and none of RCT trials, while the strength of this meta-analysis comes from the high methodological quality of each individual study as well as data homogeneity for most outcomes, including the primary outcome of proton-pump inhibitor use, complications, and adverse events (dysphagia, gas/bloating, and inability to belch and vomit).

## 5. Conclusions

Although the long-term outcomes of MSA are to be observed, GERD patients can have similar control of reflux symptoms from short outcomes, no matter what type of surgery was performed, and MSA has some advantages including shorter operative time and less complication of gas and bloating. MSA can be recommended as an alternative treatment for GERD according to their short-tern studies, especially in main-features of gas and bloating, due to shorter operative time and less complication of gas-bloating.

## Figures and Tables

**Table 1:** Baseline characteristics of studies included in the meta-analysis.

First author	Publication year	Group	Number	Sex (M/F)	Mean age (yr)	BMI	OR time	Length of stay (h)
Sheu	2015	MSA	12	7 : 5	39.3 ± 12.9	26.8 ± 4.4	63.7 ± 11.6	24.0 ± 0
		LNP	12	6 : 6	43.8 ± 9.2	26.8 ± 3.6	90.3 ± 18.0	26.4 ± 7.2
Louie	2014	MSA	34	16 : 18	54 ± 11.8	27 ± 5.1	65.3 ± 21.1	NA
		LNP	32	19 : 13	47 ± 12.2	30 ± 4.4	83.2 ± 23.4	NA
Reynolds^#^	2015							
Reynolds	2016	MSA	52	20 : 32	53	26	66 ± 23	17 ± 10
		LNP	67	36 : 31	53	27	82 ± 18	38 ± 14
Warren	2016	MSA	201	96 : 105	54 (42–64)	NA	60	13
		LNP	214	122 : 92	52 (43–64)	NA	76	32

M = male; F = female; MSA = Magnetic Sphincter Augmentation; NF = Nissen Fundoplication;

BMI = body mass index; OR time = operative time

^#^Dual studies from same author.

## Abbreviations

MSA: Magnetic Sphincter Augmentation
GERD: Gastroesophageal Reflux Disease
NF: Nissen Fundoplication.
